# Magnetic Resonance Imaging Correlates of Immune Microenvironment in Glioblastoma

**DOI:** 10.3389/fonc.2022.823812

**Published:** 2022-03-22

**Authors:** Alessandro Salvalaggio, Erica Silvestri, Giulio Sansone, Laura Pinton, Sara Magri, Chiara Briani, Mariagiulia Anglani, Giuseppe Lombardi, Vittorina Zagonel, Alessandro Della Puppa, Susanna Mandruzzato, Maurizio Corbetta, Alessandra Bertoldo

**Affiliations:** ^1^ Department of Neuroscience, University of Padova, Padova, Italy; ^2^ Padova Neuroscience Center, University of Padova, Padova, Italy; ^3^ Department of Information Engineering, University of Padova, Padova, Italy; ^4^ Veneto Institute of Oncology - Istituto di Ricovero e Cura a Carattere Scientifico (IOV-IRCCS), Padova, Italy; ^5^ Department of Surgery, Oncology and Gastroenterology, University of Padova, Padova, Italy; ^6^ Neuroradiology Unit, University Hospital of Padova, Padova, Italy; ^7^ Department of Oncology, Oncology 1, Veneto Institute of Oncology IOV-IRCCS, Padova, Italy; ^8^ Neurosurgery, Department of NEUROFARBA, University Hospital of Careggi, University of Florence, Florence, Italy; ^9^ Venetian Institute of Molecular Medicine, Fondazione Biomedica, Padova, Italy

**Keywords:** glioblastoma, tumor microenvironment, macrophages, microglia, magnetic resonance imaging, MRI

## Abstract

**Background:**

Glioblastoma (GBM) is the most commonly occurring primary malignant brain tumor, and it carries a dismal prognosis. Focusing on the tumor microenvironment may provide new insights into pathogenesis, but no clinical tools are available to do this. We hypothesized that the infiltration of different leukocyte populations in the tumoral and peritumoral brain tissues may be measured by magnetic resonance imaging (MRI).

**Methods:**

Pre-operative MRI was combined with immune phenotyping of intraoperative tumor tissue based on flow cytometry of myeloid cell populations that are associated with immune suppression, namely, microglia and bone marrow-derived macrophages (BMDM). These cell populations were measured from the central and marginal areas of the lesion identified intraoperatively with 5-aminolevulinic acid-guided surgery. MRI features (volume, mean and standard deviation of signal intensity, and fractality) were derived from all MR sequences (T1w, Gd+ T1w, T2w, FLAIR) and ADC MR maps and from different tumor areas (contrast- and non-contrast-enhancing tumor, necrosis, and edema). The principal components of MRI features were correlated with different myeloid cell populations by Pearson’s correlation.

**Results:**

We analyzed 126 samples from 62 GBM patients. The ratio between BMDM and microglia decreases significantly from the central core to the periphery. Several MRI-derived principal components were significantly correlated (p <0.05, r range: [−0.29, −0.41]) with the BMDM/microglia ratio collected in the central part of the tumor.

**Conclusions:**

We report a significant correlation between structural MRI clinical imaging and the ratio of recruited vs. resident macrophages with different immunomodulatory activities. MRI features may represent a novel tool for investigating the microenvironment of GBM.

## Introduction

Immune cell populations in the tumor microenvironment play a pivotal role in tumor progression and response to therapy (1). The landscape of the immune microenvironment of most solid tumors contains a proportion of tumor-infiltrating leukocytes located both in the center and at the invasive margin. It has been clearly demonstrated that the presence, location, and composition of the tumoral immune infiltrate may have an impact on clinical outcome (2). In many tumors, macrophages are a major component of the leukocyte infiltrate and are mainly associated with tumor growth and poor prognosis. In gliomas, they play a key role in the immune suppression of the tumor microenvironment (3–6).

Glioblastoma (GBM) is a highly aggressive primary brain tumor (7) with a median overall survival of 14.6 months (8). Despite some therapeutic options (surgery, radiotherapy, chemotherapy, and combined therapies), the prognosis is still poor. The growth of GBM alters brain tissues through different mechanisms: peritumoral edema, vascular alterations (angiogenesis and blood–brain barrier disruption), infiltration, and dislocation of peritumoral brain structures, Wallerian degeneration, glial cell activation, and leukocyte infiltration (9–12). Most immune cells within GBM are macrophages (3, 13). Resident microglial cells (MG) co-exist with bone marrow-derived macrophages (BMDM) actively recruited from the peripheral circulation (3, 5, 14). The two cell types differ not only in ontogeny but also in their functional activity, as BMDM exerts a strong immune suppressive activity (3, 5). In addition, we and others have recently demonstrated that the immune suppressive activity of BMDM changes according to the location within the tumor, increasing from the periphery to the center of the lesion (3, 15–17).

Structural magnetic resonance imaging (MRI) is routinely employed in the diagnostic work-up of GBM and, in line with the European Association of Neuro-Oncology guidelines, clinical MRI acquisition includes T2-weighted (T2w), fluid attenuation inversion recovery (FLAIR), and pre- and post-gadolinium contrast-enhanced T1-weighted (T1w, Gd+ T1w) images (18). The addition of standard diffusion imaging and a derived apparent diffusion coefficient (ADC) map (i.e., a measure of the magnitude of diffusion of water molecules within tissue) is also recommended (19).

The morphological features of the lesions, such as their location, extension, pattern of contrast enhancement, edema, and necrosis extension, are usually evaluated qualitatively from MR images. Radiomic methods or texture analyses have recently been included to obtain additional characterization of gliomas. Thus, MRI-based signatures of gliomas have been related to the histological stage (20, 21) and molecular profile (21–27) and, in a few instances, to the immune microenvironment (28–30). Non-invasive methods to characterize the GBM immune microenvironment are not currently available.

In the present study, we hypothesized that different patterns of leukocyte infiltrate may induce tissue alterations that are measurable with clinical MRI. Specifically, we tried to identify MRI correlates of the concentration of different types of myeloid cells (MG and BMDM) present in the GBM microenvironment.

We examined the preoperative conventional MRI of 62 patients with a confirmed histological diagnosis of *de novo* GBM and performed detailed immunophenotyping of the tumor microenvironment, namely, BMDM and MG identification on tumor specimens labeled intraoperatively with 5-ALA fluorescence emission. MRI feature patterns derived through principal components were related to the relative frequency of MG and BMDM from different tumor sample sites.

## Methods

### Patients

This retrospective study was conducted on a cohort of 62 patients (3) with a histologically confirmed, newly diagnosed GBM who underwent 5-aminolevulinic acid (5-ALA) fluorescence-guided first surgery at the Padova University Hospital, followed by the characterization of the leukocyte infiltrate by multiparametric flow cytometry. The inclusion criteria were: 1) a histologically confirmed, newly diagnosed GBM and 2) the availability of presurgical MRI acquisition, which had to include T2w, FLAIR, pre- and post-contrast T1w sequences, and DWI-derived ADC maps. The exclusion criteria were: GBM recurrence, MRI acquisition with a low magnetic field scanner (magnetic lower than 1.5T), lack of axial plane acquisition in at least one of FLAIR, pre- and post-contrast T1w sequences, the presence of macroscopic artifacts in MR structural images, and radiologic evidence of previous brain diagnostic or therapeutic procedures (e.g., stereotactic biopsy).

Fluorescence-guided surgery by 5-ALA is an intraoperative imaging technique that employs the intracellular accumulation of fluorescent porphyrins to differentiate between different sites according to fluorescent levels (31–33). Tissue sampling guided by fluorescence intensity allows the identification of a central necrotic area corresponding to the non-fluorescent inner layer (necrosis), a brightly fluorescent or intense area containing the majority of tumor cells corresponding to the central non-necrotic area (*core*), and a marginal area characterized by weak fluorescence intensity (margin) corresponding to the marginal area of the tumor. All surgical procedures and samplings were performed by the same experienced neurosurgeon (ADP). Tissue sampling was performed according to 5-ALA fluorescence, and no targeted-biopsies based on MRI images were performed. The ethical committee of the Veneto Institute of Oncology (Istituto Oncologico Veneto-IOV) and the Padova University Hospital approved the study protocol for tissue sampling (CESC IOV 2016/13 and 3848/AO/16, respectively) and for the retrospective analysis of the MRI data (CESC 70n/AO/20). All patients gave informed consent to tissue sampling. The study was performed in accordance with the Declaration of Helsinki and its latest amendments.

### MRI Acquisition Protocol

Clinical T1w, Gd+ T1w, T2w, FLAIR images, and ADC maps were preoperatively acquired at the Padova University Hospital on a 3T Philips Ingenia scanner for 44 of the 62 patients. For the remaining patients, analogous clinical protocols were acquired on a 1.5T Philips Achieva scanner (6 patients), a 1.5T Siemens Aera scanner (4 patients), and a 1.5T General Electric Optima_MR450w (8 patients). Additional details of the acquisition protocols are reported in **Supplementary Table 2**.

### Preprocessing of MR Images

Structural images were preprocessed prior to manually delineating the tumor area. Preprocessing included image bias field correction (34) and skull stripping (35). Structural images and ADC maps were then registered to the pre-contrast T1w of the patient using the Advanced Normalization Tools (ANTs) *antsRegistrationSyN* pipeline (36). Structural images (i.e., Gd+ T1w, T2w, and FLAIR) were linearly registered and a non-linear diffeomorphic transformation was estimated for the ADC map.

### Tumor Segmentation

Manual segmentation was performed using the ITK-Snap toolbox version 3.8.0 (www.itksnap.org) (37) slice-by-slice by a neurology resident (GS) and checked by an experienced neurologist (AS) and neuroradiologist (MA). The following areas were segmented into four regions of interest (ROI) for each tumor: necrosis, contrast-enhancing tumor (CET), non-contrast-enhancing tumor (nCET), and edema. A hemorrhage region was also detected in 5 patients. This was excluded in subsequent analyses and not taken into account. Details of the segmentation procedure are reported in **Supplementary Materials “Segmentation protocol”**.

### Imaging Features

**Figure 1:**
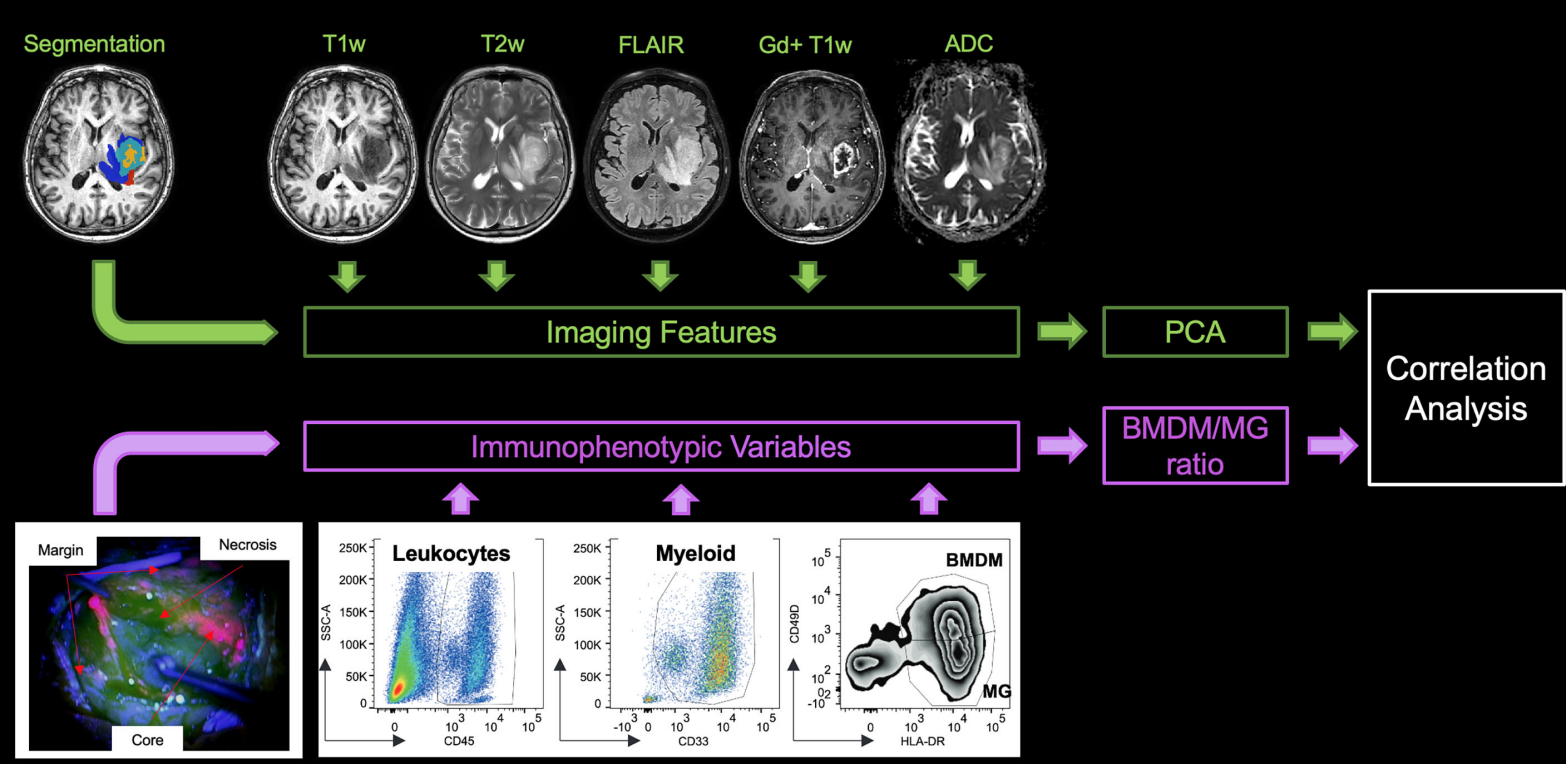
Study design. In the upper left-hand corner, the lesion segmentation superimposed onto the T1w image of the patient highlights the non-enhancing tumor core (red), the enhancing tumor core (light blue), the necrosis (yellow), and the edema (blue). At the top of the figure are the five MRI sequences from which the features were extracted. In the bottom left-hand corner, an example of the different surgical samplings according to 5-ALA fluorescence emission is shown. A representative example of the gating strategy to identify myeloid cell populations is shown in the lower part of the figure. PCA, principal component analysis, BMDM, bone marrow-derived macrophage, MG, microglia.

Features were selected to capture various phenotypic characteristics of tumor imaging at different levels of investigation and were extracted using custom codes written in Matlab (MATLAB 2020, The MathWorks, Inc., Natick, MA). Before proceeding to feature extraction, an additional post-processing step was performed on all patient images for the purposes of comparison. Images were normalized by applying a min/max normalization, using the normal-appearing area as reference tissue (i.e., outside the segmented alteration region) within the intracranial volume mask obtained in the preprocessing phase. The following quantities were computed for each patient and image: 1) the volume of the ROI and the volume of the ROI normalized to the total intracranial volume as defined by the T1w skull-stripping step (morphological features); 2) the average and standard deviation of the image intensity (intensity features); and 3) fractality (texture feature). Specifically, fractality was calculated as the average fractal dimension obtained with the modified 3D box-counting algorithm introduced by De Luca et al. (38). Morphological features were calculated for each segmentation area (i.e., two features from four areas, n = 8), whereas intensity and texture features were extracted from each image/map and for each ROI (i.e., three features from four areas for each MR image, n = 60). Overall, a total of n = 68 image features were extracted for each patient (first row, **Figure 1**).

### Tumor Sample Processing

As previously described (3), all tumor samples were processed immediately after resection. Briefly, specimens were extensively washed with 0.9% sodium chloride to remove peripheral blood and then subjected to enzymatic digestion using the human Tumor Dissociation Kit (Miltenyi Biotec, Bergisch Gladbach, Germany) and gentleMACS TM Octo Dissociator with heaters (Miltenyi Biotec), following the instructions of the manufacturer. Debris or myelin residues, if present in the cell suspension, were subsequently removed using a debris removal solution (Milteny Biotec).

### Evaluation of Myeloid Presence in GBM Tissues From Different Areas of Tumor Lesions by Multiparametric Flow Cytometry

A multiparametric phenotypic analysis was performed on 126 fresh GBM specimens collected by fluorescence-guided surgical resection from 62 GBM patients. As previously described (3), single cell suspensions obtained after specimen processing were labeled with different antibody mixtures to characterize myeloid and lymphocyte populations. Briefly, between 1 and 5 × 10^5^ cells obtained from each of the different tumor layers were washed with PBS plus 1% FBS and incubated for 10 min at 4°C with Fc-Receptor Blocking reagent (Miltenyi Biotec). The antibody mix containing anti-CD45 BV421 (BD Biosciences, Becton Dickinson, Franklin Lakes, NJ, USA), anti-CD33 PE-Cy7 (BD Biosciences), anti-HLA-DR APC (BD Biosciences), anti-CD49d PE (BioLegend, San Diego, CA, USA), and Live/Dead (L/D) Fixable Aqua (Life Technologies, Thermo Fisher Scientific, Waltham, MA, USA) as a dead cell marker, was then added to the tubes and left in incubation for 20 min at 4°C. At the end of the incubation time, cells were washed with PBS plus 1% FBS and samples were acquired by a flow cytometer. Each specimen was characterized for the presence of leukocyte infiltrate (CD45^+^ cells among live cells), myeloid cells (CD33^+^/CD45^+^ cells), BMDM (CD45^+^/CD33^+^/HLA-DR^+^/CD49d^+^), and MG cells (CD45^+^/CD33^+^/HLA-DR^+^/CD49d^−^) as shown in **Figure 1**. Data were acquired using a LSRII flow cytometer (BD Biosciences) equipped with 4 lasers (405, 488, 561, and 640 nm) and an analysis was performed by means of FlowJo software v10.4 (Becton Dickinson, Franklin Lakes, NJ, USA). All antibodies used for flow cytometry were titrated in a batch-dependent manner.

### Statistical Analyses

MRI imaging and immunophenotypic variables were first evaluated separately in the four ROIs (i.e., *necrosis*, *nCET*, *CET*, and *edema*) or sample sites (i.e., *necrosis*, *core*, and *margin*). Then, we checked for a significant relationship between these two groups of features.

We ran a principal component analysis (PCA) on the MRI imaging variables to investigate their collinearity and reduce their dimensionality. Each imaging feature was z-scored across the group, and the PCA was run on the 17 imaging features selected *a priori* (i.e., for each ROI: two morphological features, plus three intensity or texture features for each MR image) separately on each of the four pathological tissues (i.e., *necrosis*, *nCET*, *CET*, and *edema*). We also ran a PCA that took into account imaging features across all types of tissue (*all*). Next, to investigate whether the imaging features (expressed as principal components [PCs]) conveyed the same information across pathological tissues, we first ran a Pearson’s correlation of the first four PCs (cumulatively explaining 78% of the total variance), then we quantified the similarity of the PC loadings across tissues, using the structural similarity index computed between pairs of loading matrices.

We applied a similar approach to the immune parameters, namely BMDM, MG, myeloid cells (myeloid), and leukocyte infiltrate (leukocytes). First, the distribution of each variable was compared across sampling sites (i.e., *necrosis*, *core*, and *margin*) using the non-parametric Kruskal–Wallis and Mann–Whitney tests (significance threshold set to 0.05). To investigate the collinearity of the variables and reduce their dimensionality, each immunological feature was z-scored across the group and a multi-group PCA (39) was run taking into account the samples derived from all the 3 sites together (*all*, i.e., considering the sample from any site without topographical distinction; if more than one sample was collected for a subject, the mean value was considered) or separately by considering the samples derived from each specific site (*necrosis*, *core*, and *margin*). A structural similarity index was computed between pairs of loading matrices to evaluate whether immunophenotypic variables were similarly related across the four sampling sites.

Given that the variance for all sampling sites was mainly explained by the first component, and in this component the BMDM and MG variables were the most representative in terms of loadings, a novel index was introduced to summarize the immunophenotypic variables, namely the BMDM/MG ratio. This index was computed independently for each sampling site. To assess the sensitivity of this index in differentiating between different sampling sites, its distributions across sampling sites were compared using the Kruskal–Wallis and Mann–Whitney tests (significance threshold set to 0.05).

Finally, the relationship between imaging (expressed as PCs) and immunophenotypic features was explored for each pathological tissue using a Pearson’s correlation between the BMDM/MG ratio of each imaging sample site (**Figure 1**).

### Code Availability

The data and codes that support the findings of this study are available from the corresponding author on reasonable request.

## Results

### Patient Characteristics

The final study cohort consisted of 62 GBM patients who underwent preoperative and conventional Gadolinium-enhanced MRI, followed by fluorescence-guided resection with 5-ALA-assisted surgery for GBM resection between 2016 and 2019. The study population included 44 males and 18 females (mean age 61.9 ± 10.9 y) with a total of 126 specimens analyzed, derived from *core*, *necrosis*, and *margin* areas (58, 37, and 31, respectively). In 46 cases, multiple specimens were analyzed, corresponding to different tumor areas characterized by high (*core*), weak (*margin*), or absent (*necrosis*) fluorescence intensity. The demographic data and clinical variables of the study cohort are reported in **Table 1**.

**Table 1:** Demographic data and clinical variables of the study cohort.

	N*	Sex (M/F)	Age at surgery (mean and SD) yrs	Days between MRI and surgery (median and quartile)	IDH1 (wt/mutant/n.a.)	MRI magnetic field strength (3T/1.5T)
All subjects	62	44/18	61.9 (10.9)	8.5 (4.3–15.5)	60/1/1	44/18
Subjects with ALA-intense (*core*) samples	58	42/16	61.7 (11.0)	8 (4–16)	56/1/1	42/16
Subjects with *necrosis* samples	37	23/14	64.6 (9.6)	9 (5–17)	35/1/1	23/14
Subjects with *margin* area samples	31	18/13	63.1 (12.5)	11 (6.5–19.5)	29/1/1	21/10

*A subject may have more than one sampling site.

N, number of subjects; Wt, wild type; n.a., data not available; SD, standard deviation; M, male; F, female; T, Tesla.

### Multimodal MRI-Derived Features

**Figure 2:**
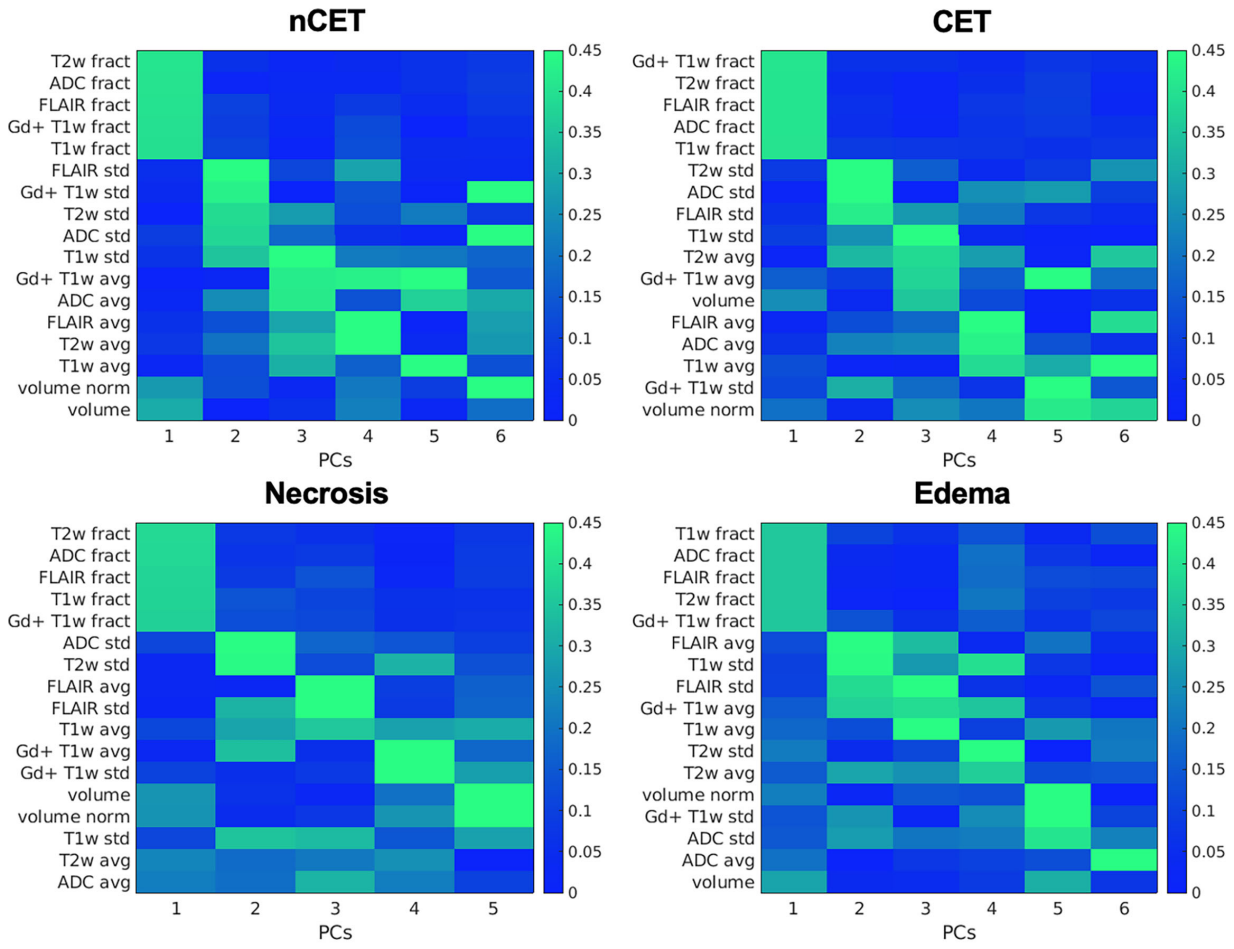
PCA on imaging features: PC loadings are reported explaining 80% of the variance. Variables are ordered (y-axis) according to their relative loadings for each PC. Loadings are reported separately for each tissue. *nCET*, non-contrast-enhancing tumor; *CET*, contrast-enhancing tumor; *PCs*, principal components; *fract*, fractality; *std*, standard deviation; *avg*, average; *norm*, normalized for the total tumor volume.

**Figure 3:**
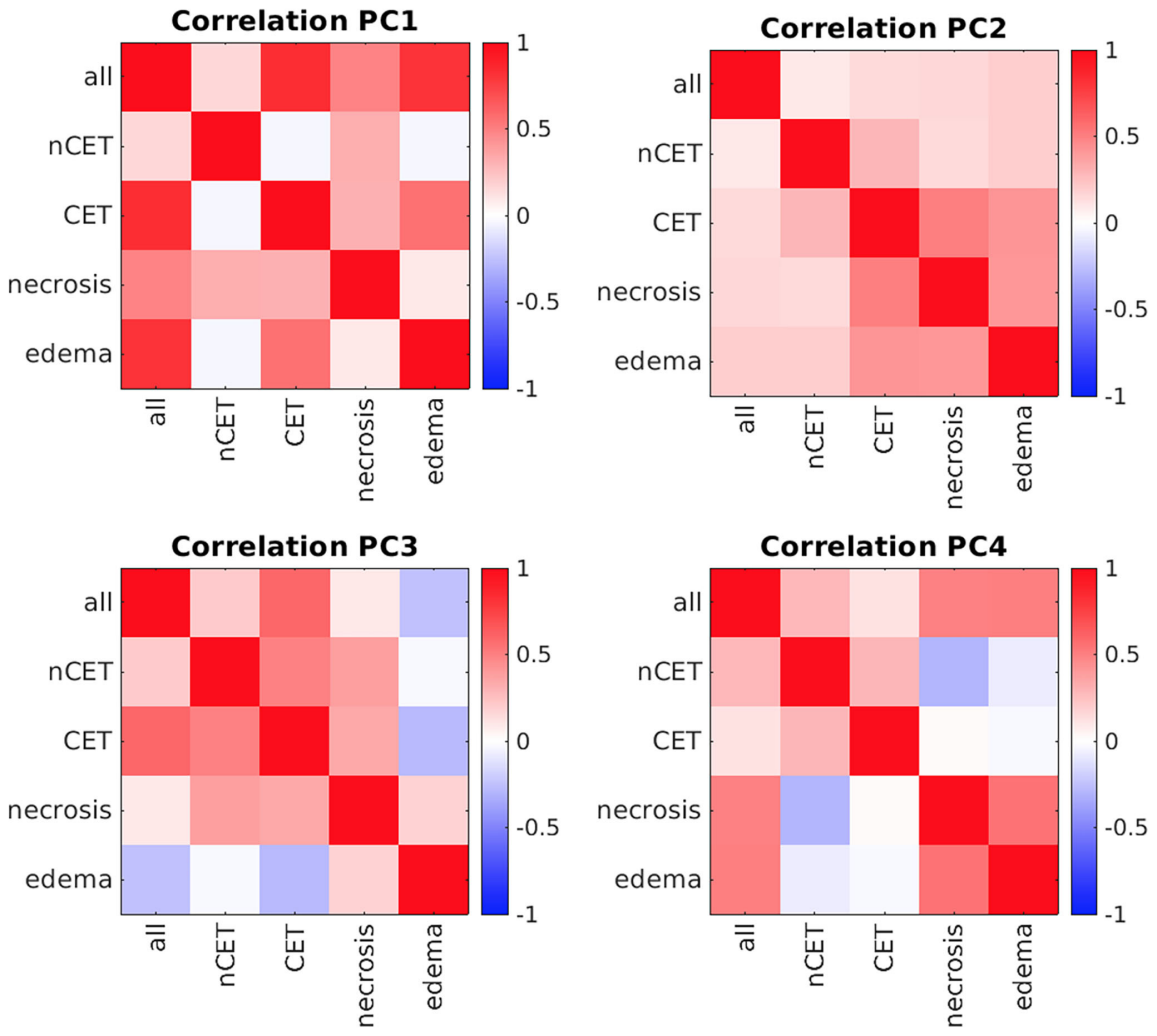
Correlation matrixes between principal components (PCs) across tissues. Positive correlations are represented in red, while negative correlations are in blue. *All*, whole tumor including edema; *nCET*, non-contrast-enhancing tumor; *CET*, contrast-enhancing tumor.

Patients underwent preoperative MRI and, for the purpose of this study, four ROIs were determined in the tumor lesion (nCET, CET, necrosis, and edema), as detailed in the **Supplementary Materials Segmentation protocol**. The mean overall volume of the lesions was 95.3 cm^3^ ( ± 49.9); the mean volume of nCET was 9.0 cm^3^ ( ± 9.7); the mean volume of CET was 23.0 cm^3^ ( ± 16.5); the mean volume of necrosis was 17.0 cm^3^ ( ± 18.9); and the mean volume of edema was 46.7 cm^3^ ( ± 37.4). Following the segmentation of MRI images, the PCA was run separately on the set of 17 features extracted from each of the four pathological tissues. The number of principal components (PCs) that describe at least 80% of the total variance was 5 for the necrotic tissue and 6 for the others. The mean variance explained by the first PC of the four tissues was 36.6% (range 33.7–41.5%). As shown in **Figure 2**, the first PCs exhibited the same magnitude and pattern of loadings across the four tissues. The first PC was largely explained by the fractality of T2w, ADC, FLAIR, Gd + T1w, and T1w. Interestingly, this pattern was the same across the four tissues. The second PC was mainly expressed by standard deviation, while from the third PC, the pattern of loadings lost their correspondence to the single features. When the features from the four tissues were considered in aggregate (n = 17 × 4), fractal loadings were distributed among the first two PCs, accounting for 32.0% of the total variability (**Supplementary Materials Figure 1**). From the third component onwards, any additional specific feature and/or imaging modality and/or tissue was not clearly discernible, even if the loadings of the third component were mainly expressed by standard deviation. Notably, the PC loadings seem to depend on the type of features (fractality, SD, and average) rather than the image (T1w, Gd+ T1w, T2w, FLAIR, or ADC). The structural similarity index between the PC loadings of the four tissues (i.e., *nCET, CET, necrosis*, and *edema*) showed high dissimilarity across the different tissues. The highest value was 0.57 (range 0–1, with 1 = complete agreement) between edema and necrosis; the lowest was 0.23 between nCET and edema. Poor matching also appears when looking at the correlation matrixes in **Figure 3**, where, starting from PC2, the PC loadings diverged (i.e., low correlation) across the four tissues. Moreover, PCs obtained from aggregate tissue (17 × 4) are different to the PCs obtained from each single tissue (except for the first PC). Redundancy of information across tissues was excluded based on these results. Therefore, immune-MRI correlates were investigated on the imaging features of each of the four tissues (separately) and aggregate tissues.

### Evaluation of Myeloid Cell Presence in GBM Tissues From Different Tumor Lesion Areas

Sixty-two GBM patients underwent surgical excision of the lesion using an intraoperative navigation system, which used 5-ALA fluorescence to identify three different tumor areas characterized by high (*core*), dim (*margin*), or absent (*necrosis)* fluorescence intensity. Where possible, three tumor specimens from each patient were collected from the *necrosis, core*, and *margin*. In some instances, only specimens from one or two areas were obtained. Out of 62 patients, 58, 37, and 31 specimens were collected from the *core, necrosis*, and *margin* areas, respectively, for a total of 126 tumor specimens (**Table 1**). Multiparametric flow cytometry was used to characterize the presence of leukocyte infiltrate (CD45^+^ cells among live cells), myeloid cells (CD33^+^/CD45^+^ cells), BMDM (CD45^+^/CD33^+^/HLA-DR^+^/CD49d^+^), and MG cells (CD45^+^/CD33^+^/HLA-DR^+^/CD49d^−^) in each specimen, as shown in **Figure 1**.

**Figure 4:**
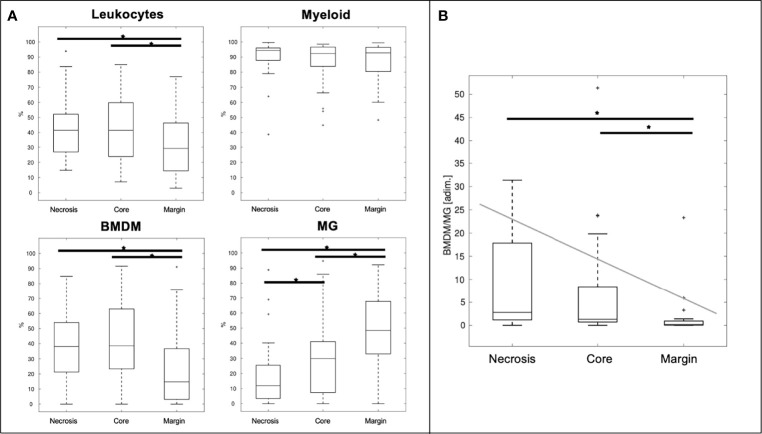
Distributions of the investigated immune populations **(A)** and ratio between BMDM and MG **(B)** at different sampling sites. * = significant differences (p < 0.05), leukocyte infiltrate = CD45^+^ cells gated inside live cells; myeloid = CD33^+^ cells gated among CD45^+^ cell; BMDM = bone marrow-derived macrophages HLA-DR^+^/CD49d^+^ cells gated inside myeloid cells; MG = microglia HLA-DR^+^/CD49d^-^ cells gated inside myeloid cells.

As shown in **Figure 4**, a median of 41.5% of live cells, obtained from the dissociation of necrosis and *core* regions, are infiltrating leukocytes. This percentage is reduced to 29.2% in the marginal area (**Figure 4A**, upper left panel). Myeloid cells make up more than 90% (94.3, 92.3, and 92.8% of *necrosis*, *core* and *margin* areas, respectively) of the leukocyte infiltrates (CD45^+^ cells) (**Figure 4A**, upper right panel). As regards myeloid cells, the presence of MG was significantly higher in the marginal area compared to the central *core* area and the necrotic area (p-value <10^−3^ and p-value <10^−5^, respectively). In contrast, the frequency of BMDM and leukocyte infiltrates was significantly lower in the marginal area than in the central area, including necrosis and *core* sampling (p-value <10^−2^ in all cases), in line with our previous results (3). Overall, the BMDM/MG ratio decreases significantly from the central area to the periphery of the tumor (**Figure 4B**). Based on these results, the BMDM/MG ratio was chosen to describe the immunological gradient within the GBM microenvironment, and it was adopted as the immunological biomarker to correlate with MRI feature PCs, as displayed in **Figure 1**.

### PCA With Immune Variables Derived From Different Sampling Sites

We ran a PC analysis on the leukocyte, myeloid, BMDM, and MG immune parameters. The first two PCs described at least 80% of total variance in all tissues, with the exception of necrosis, where 3 PCs were necessary to explain the same level of variance. The mean variance explained by the first PC across the four tissues was 58.3% (range 50.5–62.4%). In contrast to the imaging features, PC loadings for the immune variables showed a consistent pattern (both in magnitude and direction) across the different sampling sites (**Supplementary Materials Figure 2**), except for the reverse direction of BMDM and MG in the first component of the marginal area. Accordingly, the structural similarity index for *core*, *necrosis*, and *all* types of tissue in aggregate was high, ranging from 0.66 to 1 (i.e., considering the sample from any site without topographical distinction). Low similarity (range 0.16–0.28) was found in the samples on the marginal area. These findings suggest that immunological information is insufficient to distinguish between the different regions of the tumor, since the PCA patterns of the four cell types (leukocytes, myeloid, BMDM, and MG) were almost the same in the *necrosis*, *core*, and *all* sites, despite the emergence of some significant differences when comparing the distribution of the single immune populations across the different tissues (**Figure 4A**). Subsequently, we evaluated the combination of two immunological variables by using their ratio value (i.e., the BMDM/MG ratio) to improve the ability to distinguish of the immunological information between the three sampling sites. In fact, the BMDM/MG ratio can describe the immunological gradient within the GBM microenvironment, and it was adopted as the immunological biomarker to correlate with the MRI feature PCs (**Figure 4B**).

### Correlation Between the Presence of Tumor Macrophages in Different Tumor Areas and MRI-Derived Features

**Figure 5:**
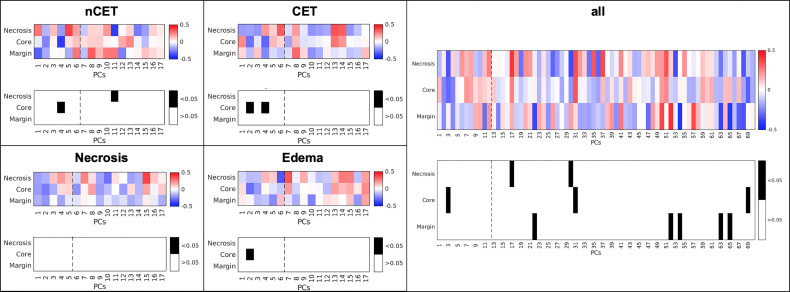
Correlation between MRI-derived PCs and the BMDM/MG ratio in different sampling sites and in different MRI-derived tissues. The dotted line represents 80% of variance explained by PCs. *nCET*, non-contrast-enhancing tumor, *CET*, contrast-enhancing tumor, *PCs*, principal component.

The potential correlation between MRI-derived features and the presence of myeloid cells was investigated by considering, on the one hand, the BMDM/MG ratio and, on the other, the imaging PCs from each of the four tissues, both separately and for the whole tumor, i.e., the area delineated by all four tissues. The BMDM/MG ratio in the *core* area correlated negatively with PC4 of nCET (R = −0.41, p = 0.002), with PC2 and PC4 of CET (R = −0.29, p = 0.04, and R = −0.31, p = 0.02, respectively), and with PC2 of edema (R = −0.29, p = 0.03) (**Figure 5**). No correlation was observed for MRI-derived features measured within *necrosis*. When considering the whole tumor, the only significant correlation was in the *core* area with PC3 (R = −0.30, p = 0.02) (**Figure 5**). The BMDM/MG ratio correlated with PC loading on the SD and average values of different MR images (i.e., intensity features) within *nCET*, *CET*, and *edema*. Other correlations emerged for higher PCs beyond the 80% variance explained, but their significance is doubtful.

As the BMDM/MG ratio describes the extent of blood macrophage recruitment, which is mainly responsible for the immune suppressive microenvironment, our results demonstrate that the infiltration of blood-derived macrophages has an impact on some MRI parameters in the same area in which these cells were found, and in the marginal area of the tumor. These results suggest that a radiomic profiling of GBM has the potential to characterize the heterogeneity of the suppressive microenvironment.

## Discussion

The present study examined the correlations between clinical MRI and the immune microenvironment in GBM. Our aim was to identify a non-invasive technique able to analyze the presence and composition of the GBM immune infiltrate, given its role in tumor progression. The underlying assumption was that the immune infiltrate can shape tumoral–peritumoral tissue and that its presence can be detected by one or more MRI features, notwithstanding the large spatial scale differences between *in vivo* imaging and the immune microenvironment.

Our study not only demonstrated that MRI-derived measures are significantly associated with the presence of tumor macrophages (evaluated as a ratio between recruited and resident cells), but that this association depends on macrophage localization in different tumor areas defined by MRI parameters and by fluorescent-dependent localization. Moreover, the BMDM/MG ratio emerged as the best feature to characterize the immune-suppressive microenvironment. Given the predominance and the large presence of tumor macrophages in GBM lesions, it is worth noting that the BMDM/MG ratio was also correlated with some multimodal MRI features.

In terms of anatomical correlation between tumor location defined by 5-ALA fluorescence emission and tumor segmentation, as assessed by preoperative MRI, we and others have previously reported that the two techniques are complementary, but not entirely overlapping. In fact, by analyzing the tumor from the inner to the marginal region, it was observed that the CET region matches the bright fluorescent area (core), and that the MRI FLAIR alteration may correspond to the weak fluorescent marginal area. However, weak fluorescence may also be detected in edema or normal-appearing tissue at MRI (33, 40, 41). In addition, we recently demonstrated that protoporphyrin IX (PpIX) fluorescent emission, the main fluorescent metabolite derived after 5-ALA administration, is not entirely dependent on GBM tumor cells, but it also identifies immunosuppressive BMDM present in GBM tissues (3, 42).

We investigated the high heterogeneity of GBM tissue through a PCA approach to reduce the dimensionality of many MRI variables, namely, sequences, features (e.g., fractality), and four tumor areas (necrosis, CET, nCET, and edema). The low similarity of PC loadings across the four tumor areas shows that tissues derived from different locations of the same tumor provide additional information, hence suggesting that the four-tissue segmentation of GBM should be considered the state-of-the-art in imaging studies (43). The first two PCs are related to features (e.g., fractality and standard deviation) that reflect the variability of the image signal within the tumor across different sequences, as compared to volume or mean signal intensity. This suggests that indices of image signal variability represent a reliable and easy to assess biomarker of GBM tissue heterogeneity.

The four immune populations were likewise investigated with PCA. The high similarity index between PC loadings across the different sample sites (*necrosis*, *core*, and *margin*) indicated a redundancy in the information. However, the two macrophage subsets (BMDM and MG) detected by flow cytometry showed an opposite gradient within the tumor, with BMDM decreasing from the central to the marginal area and MG cells increasing from the *necrosis* and *core* to the *margin*. Therefore, the BMDM/MG ratio was introduced as a new immunological index. The opposite gradient reflects the immunological properties of these two cell types, as BMDM prevents the immunological response of MG to tumor cells. Therefore, the BMDM/MG index represents a functional parameter in which a higher value corresponds to a microenvironment permissive to tumor growth.

It is interesting to note that there was a significant correlation between imaging data and the BMDM/MG index only within ALA-intense (*core*) sampling. Therefore, imaging PCs from different tissues correlate with the immune microenvironment only in a specific site—the highly fluorescent *core* region. These findings suggest that the immune microenvironment within an ALA-*intense* area has an impact beyond the border of that specific location, while the BMDM/MG ratio measured in other sampling areas is not detectable by MRI. These findings are consistent with the fact that leukocyte populations infiltrating GBM differ not only in quantity but also in their activity across different tumor locations (3, 14–16, 44).

An additional note regards the loadings of the imaging PCs showing significant correlations with the immunosuppressive cells. Their values (either positive or negative) indicate that T2w and Flair images have the most prominent role in detecting the relationship between the PCs and the immune variables. In fact, through the two sites of sampling (*core*, *intense ALA*), larger loadings are more frequently associated to T2w and FLAIR average and standard deviation values obtained in nCET, CET and Edema areas (**Supplementary_Materials Figures 9**–**11**). Interestingly, both MR images are related to the magnetic relaxation property of the tissues and sensitive to the presence of intracellular iron stores. Iron deposition has also been linked to neurodegeneration and inflammation (45). This suggests a possible role of iron in the quantitative description of the immunosuppressive environment in glioblastoma.

The present study has some limitations: i) The potential role of MRI-derived and immunological features as prognostic factors was not investigated; ii) a cross-validation approach by splitting the data between training and validation sets was not performed, given the dimension of the dataset; iii) MR images were obtained with different scanners and with sequences that were not harmonized, with the potential result of a significant between-feature variability; and iv) the a full clinical MRI acquisition (i.e. T1w, T2w, FLAIR, Gd+ T1w and ADC) was not performed on the same day of the surgery and, consequently, of the tumor sampling.

In conclusion, the present study demonstrates a significant correlation between pre-surgical MRI and the presence of recruited and resident macrophages in the tumor microenvironment endowed with different immunomodulatory activities. Given the role of these macrophages in tumor progression, the monitoring of these parameters may represent a novel tool for investigating the tumor microenvironment.

## Data Availability Statement

The raw data supporting the conclusions of this article will be made available by the authors, without undue reservation.

## Ethics Statement

The studies involving human participants were reviewed and approved by the ethical committee of the Venetian Oncologic Institute (Istituto Oncologico Veneto-IOV) and the Padova University Hospital. The patients/participants provided their written informed consent to participate in this study.

## Author Contributions

AS, ES, SuM, MC, and AB designed the study. LP and SaM performed the flow cytometry experiment and analyzed data. GS, AS, and MA generated the lesion masks. AS, ES, GS, and AB analyzed the imaging data and performed the methods for their integration with immunophenotyping data. AS, ES, LP, CB, GL, VZ, ADP, SuM, MC, and AB participated in the interpretation of data. AS, ES, SuM, MC, and AB wrote the manuscript. All authors listed have made a substantial, direct, and intellectual contribution to the work and approved it for publication.

## Funding

SuM was supported by the TRANSCAN-2, ERA-NET, IMMUNOGLIO Università degli Studi di Padova (DiSCOG Department, grants number BIRD205873/20 and BIRD188051/18), the Ministero della Salute (RF-2019-1236925), and the IOV-IRCCS (BIOV19MANDR). MC was supported by the MIUR Departments of Excellence Italian Ministry of Research (MART_ECCELLENZA18_01); the Fondazione Cassa di Risparmio di Padova e Rovigo (CARIPARO) Ricerca Scientifica di Eccellenza 2018 (Grant Agreement number 55403); and the Celeghin Foundation Padova (CUP C94I20000420007).

## Conflict of Interest

The authors declare that the research was conducted in the absence of any commercial or financial relationships that could be construed as a potential conflict of interest.

## Publisher’s Note

All claims expressed in this article are solely those of the authors and do not necessarily represent those of their affiliated organizations, or those of the publisher, the editors and the reviewers. Any product that may be evaluated in this article, or claim that may be made by its manufacturer, is not guaranteed or endorsed by the publisher.
