# Supplementary material for: Magnetic Resonance Imaging Correlates of Immune Microenvironment in Glioblastoma
Source: Front Oncol. 2022 Mar 22;12:823812. doi: 10.3389/fonc.2022.823812 (PMC8980808; doi:10.3389/fonc.2022.823812)
Supplement: Supplementary file 1 [file DataSheet_1.pdf]

Supplementary Material

1.1 Supplementary Figures

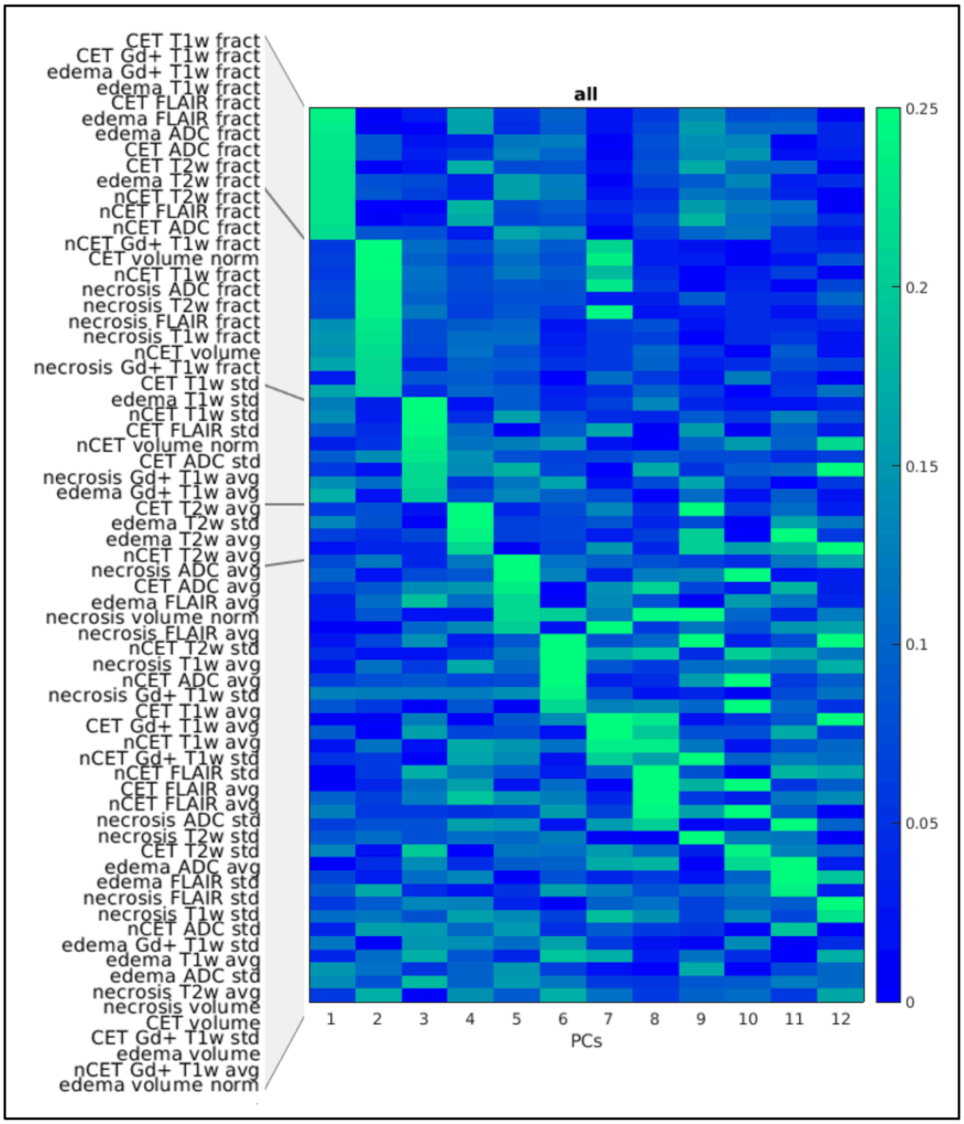

Supplementary Figure 1: The PCA on all imaging features: loadings of the PCs explaining the 80% of the variance are reported. Variables are ordered (y-axis) according to their relative loadings for each PCs. Grey lines highlight the set of imaging features that have the highest weight in each of the first four PCs separately considered. *fract = fractality, std = standard deviation, avg = average, norm = normalized for the total tumor volume.*

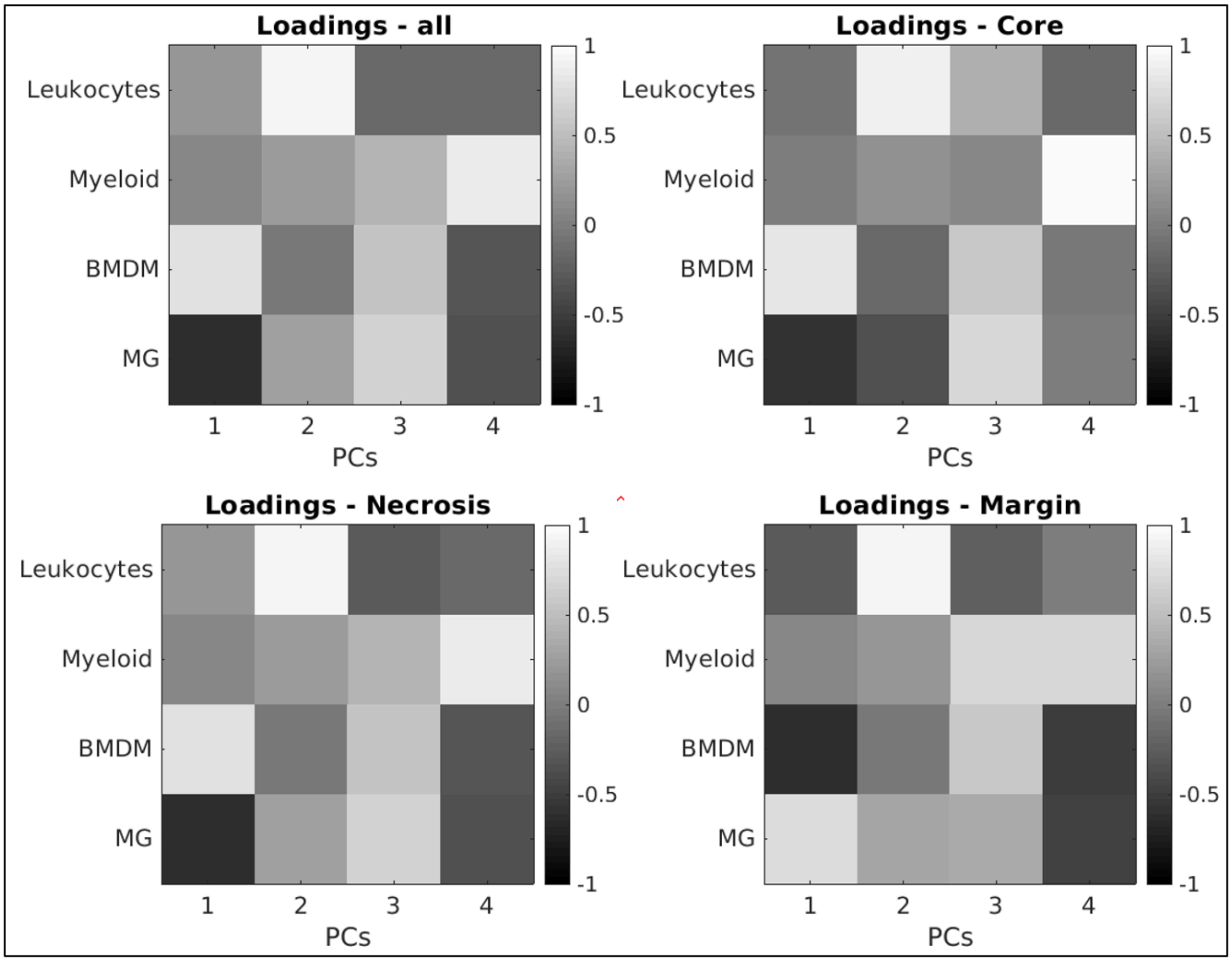

Supplementary Figure 2: Loadings of PCA run on immunological features separately considering the four sampling sites (i.e., all tissues, core, necrosis and margin).

Segmentation protocol

Manual segmentation was performed with the ITK-Snap toolbox version 3.8. (www.itksnap.org) slice-by-slice. Structural images (i.e., Gd+ T1w, T2w, and FLAIR) were linearly registered (whereas a non-linear diffeomorphic transformation was estimated for the ADC map). By setting the opacity of the different images, overimposed on each other, segmentation was therefore performed taking in account at the same time the signal intensity across all the images. Morevoer, morphological features of the tumor were considered.

Here an example from the study cohort of step by step segmentation:

STEP 1: visualisation of pre-contrast T1, coregistered post-contrast T1 and T2-FLAIR images.

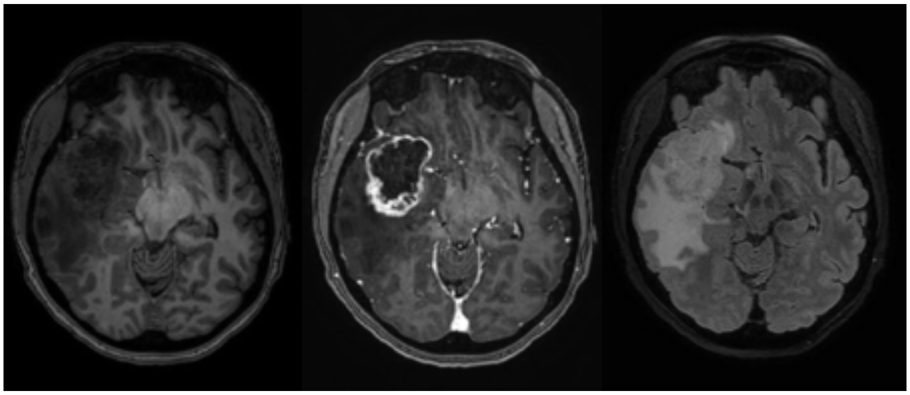

Supplementary Figure 3: axial pre-contrast T1 (left)), post-contrast T1 (middle) and T2-FLAIR (right) sections of a GBM

STEP 2: contrast-enhancing tumour (CET) segmentation using post-contrast T1 sequences

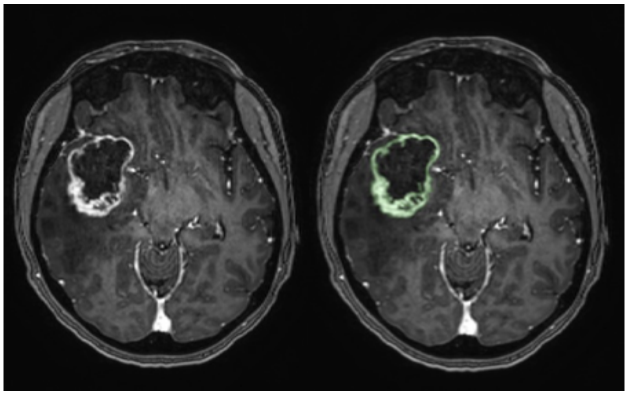

Supplementary Figure 4: axial post-contrast T1 section before(left) and after (right) CET segmentation (green), in transparency

STEP 3: **necrosis segmentation using post-contrast T1 sequences. Typically, necrosis is enclosed by CET.**

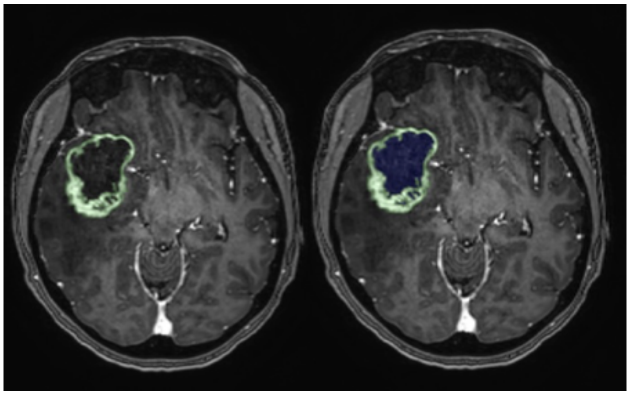

Supplementary Figure 5: axial post-contrast T1 section with CET segmentation, before (left) and after (right) necrosis segmentation label (blue), in transparency

STEP 4: edema segmentation using T2-FLAIR sequences.

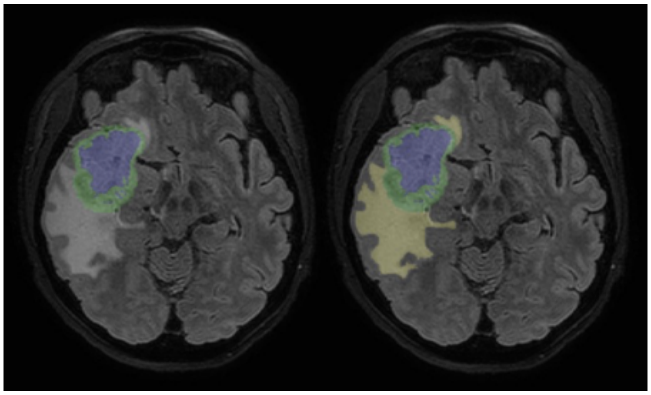

Supplementary Figure 6: axial T2-FLAIR section with CET and necrosis segmentations, before (left) and after (right) edema segmentation (yellow), in transparency

STEP 5: non contrast-enhancing tumour (nCET) segmentation using T2-FLAIR sequences.

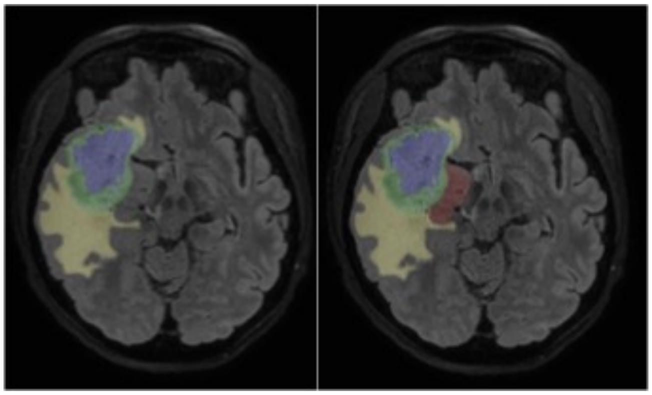

Supplementary Figure 7: axial T2-FLAIR section with CET, necrosis and edema segmentations, before (left) and after (right) nCET segmentation (red), in transparency

All the abovementioned steps were carried out throughout the whole tumour volume, using all MR sections showing GBM. Below*, figure 8* shows different sequences used, with the superimposed complete segmentation.

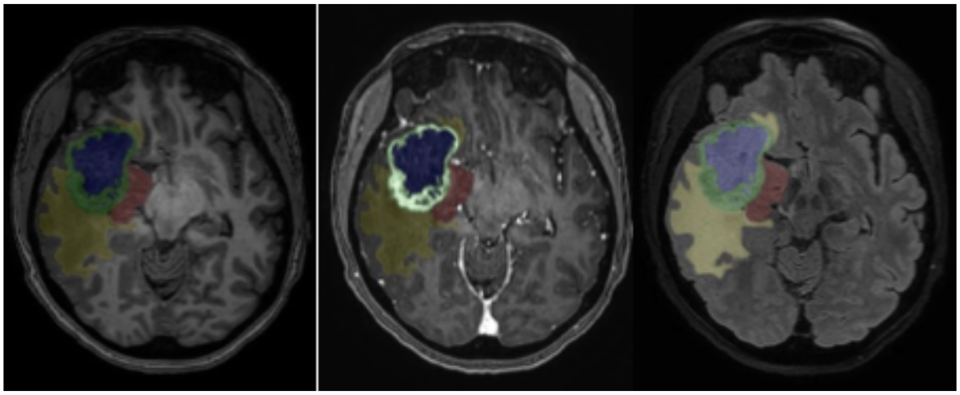

Supplementary Figure 8: axial pre-contrast T1, post-contrast T1, T2-FLAIR sections with all segmentation labels.

To discriminate between edema and nCET, the following criteria (*Supplementary Table 1)* were used.

	**Conventional MRI features useful for identifying <u>edema</u>**[1]	**Conventional MRI features useful for identifying <u>non contrast-enhancing tumour</u>**[1,2]
**Morphologic criteria**	"Finger-like appearance", confined to the white matter<br><br>Mostly concentric around CET	Extension beyond CET margin with an eccentric appearance
**White/grey matter involvement**	Sparing of both cortical and basal nuclei grey matter, possible extension along the internal or external capsule	Involvement of both white and grey matter: typical examples are cerebral cortex, basal nuclei, corpus callosum
**Mass-effect**	Diffuse or generalised mass-effect	More subtle and localised mass effect, causing architectural distortion. Such anatomical deformations become evident when compared to contralateral intact structures
**T2-FLAIR intensity**	Marked T2 FLAIR hyperintensity, often fading towards the periphery of edema	Relatively mild T2 FLAIR hyperintensity, compared to edema: this is coherent with the recent finding that T2 FLAIR signal intensity is inversely correlated to cellularity.[3]

Supplementary Table 1

Supplementary References

1. Lasocki, A. & Gaillard, F. Non-Contrast-Enhancing Tumor: A New Frontier in Glioblastoma Research. *Am. J. Neuroradiol.* **40**, 758–765 (2019).

2. Lasocki, A., Gaillard, F., Tacey, M., Drummond, K. & Stuckey, S. Morphologic patterns of noncontrast-enhancing tumor in glioblastoma correlate with IDH1 mutation status and patient survival. *J. Clin. Neurosci.* **47**, 168–173 (2018).

3. Chang, P. D. *et al.* A multiparametric model for mapping cellularity in glioblastoma using radiographically localized biopsies. *Am. J. Neuroradiol.* **38**, 890–898 (2017).

Loading of the PCs correlated with the BMDM/MG ratio in the three sampling sites

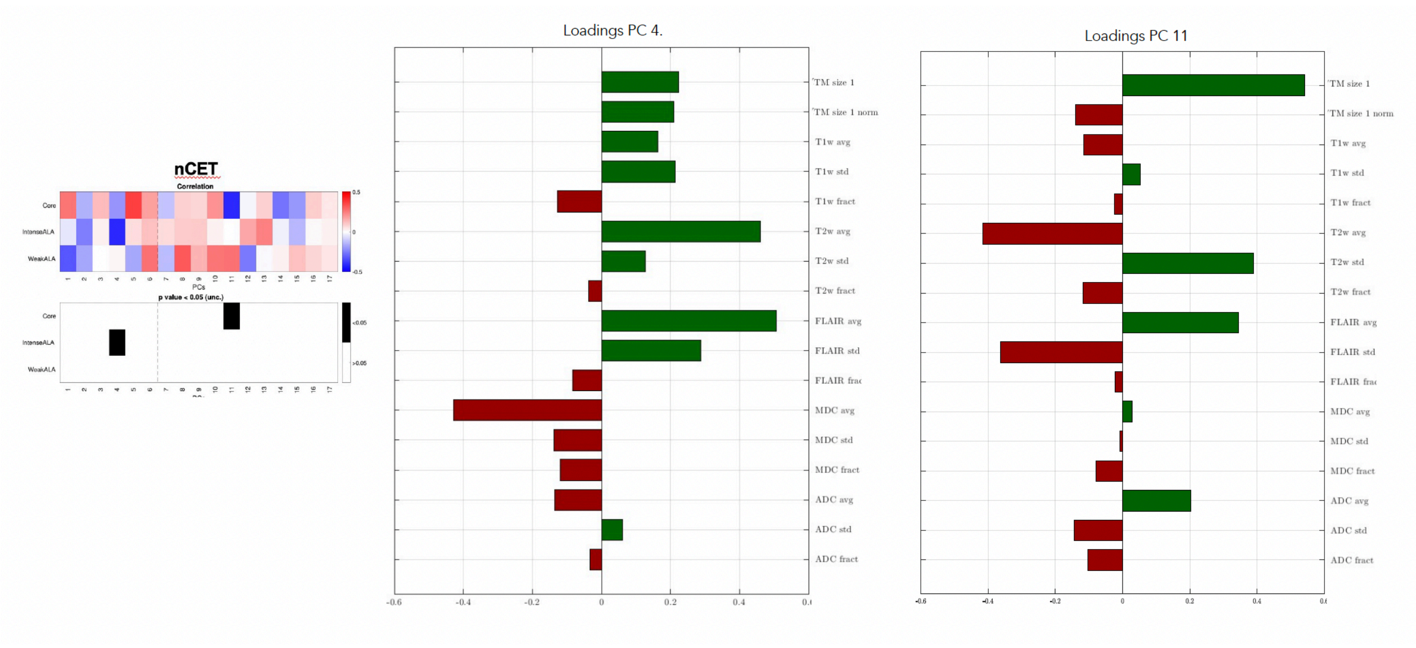

Supplementary Figure 9: *Loadings of PC4 and PC11 obtained from nCET imaging feature analysis*

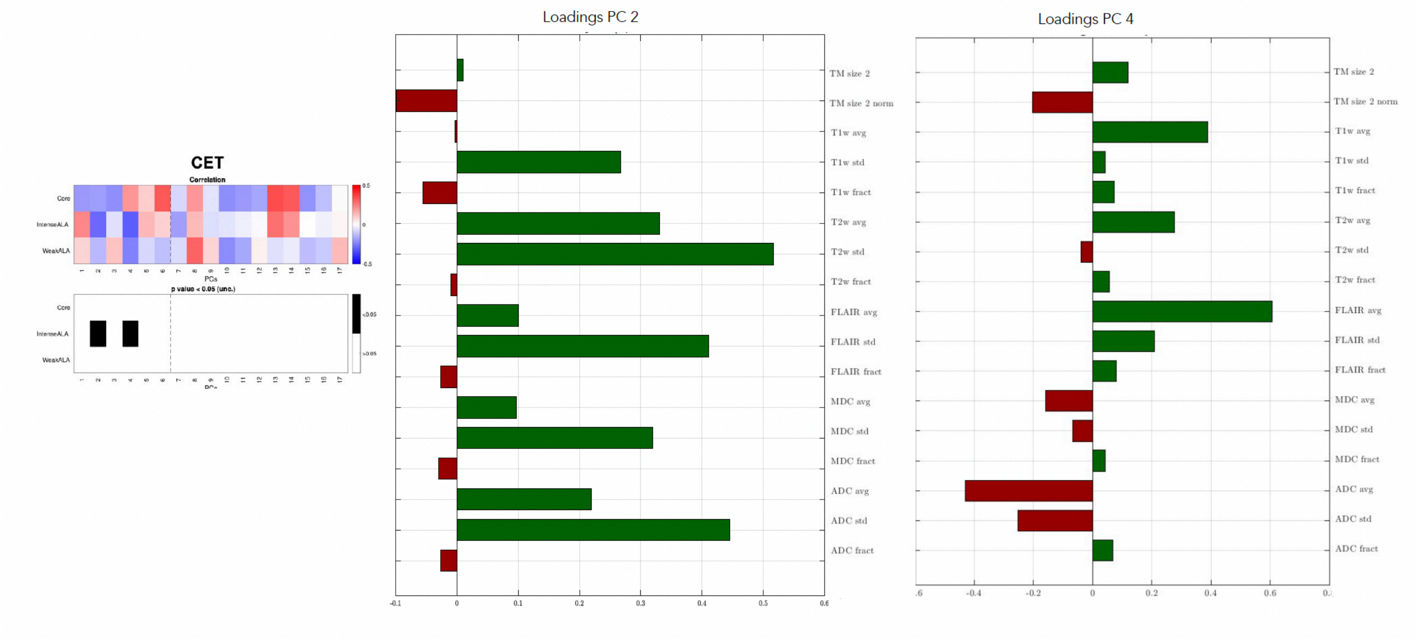

Supplementary Figure 10: *Loadings of PC2 and PC4 obtained from CET imaging feature analysis*

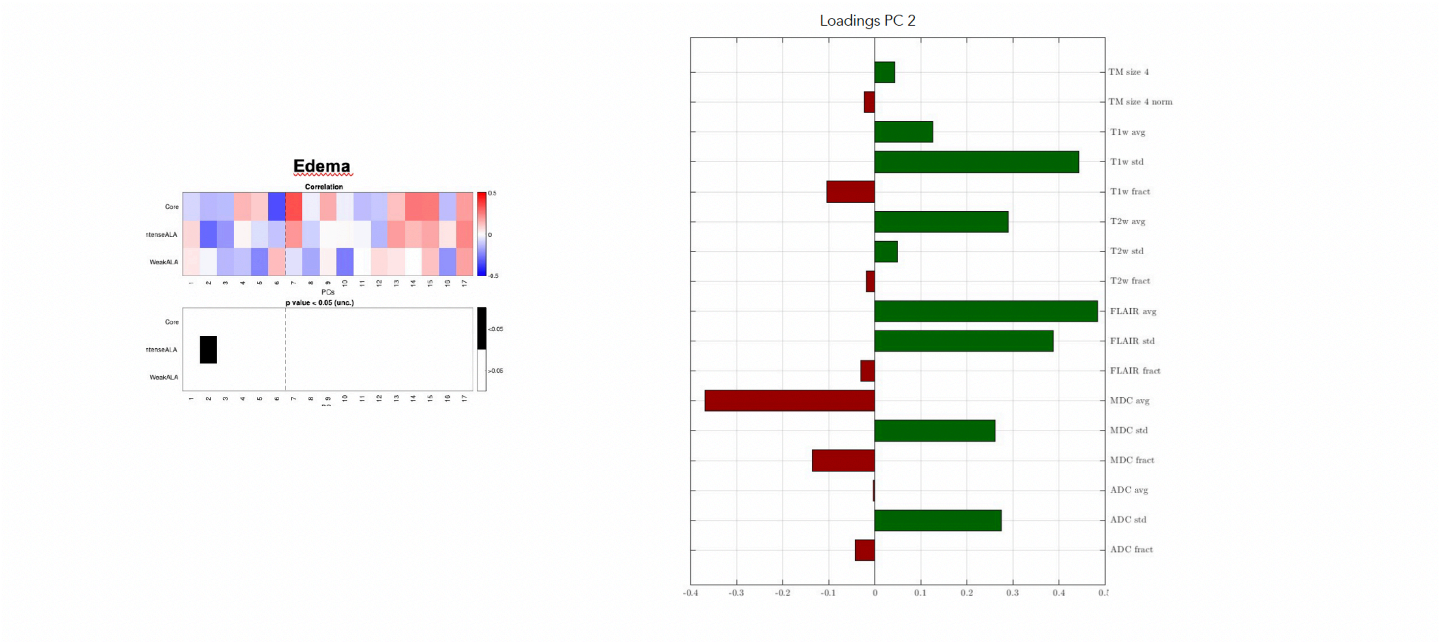

Supplementary Figure 11: *Loadings of PC2 obtained from Edema imaging feature analysis*
